# Bilateral Sensorineural Deafness in a Young Pregnant Female Presenting with a Fever: A Rare Complication of a Reemerging Disease—Spotted Fever Group Rickettsioses

**DOI:** 10.1155/2019/5923146

**Published:** 2019-03-25

**Authors:** A. G. T. A. Kariyawasam, D. R. Palangasinghe, C. L. Fonseka, P. U. T. De Silva, T. E. Kanakkahewa, N. J. Dahanayaka

**Affiliations:** ^1^Registrar in Medicine, University Medical Unit, Teaching Hospital Karapitiya, Galle, Sri Lanka; ^2^Consultant Physician, University Medical Unit, Teaching Hospital Karapitiya, Galle, Sri Lanka; ^3^Consultant Physician, Department of Internal Medicine, Faculty of Medicine, University of Ruhuna, Matara, Sri Lanka; ^4^Senior Registrar in Medicine, University Medical Unit, Teaching Hospital Karapitiya, Galle, Sri Lanka

## Abstract

**Background:**

Rickettsial illnesses are a group of arthropod-borne remerging diseases. They are subdivided into three groups as typhus, spotted fever, and scrub typhus group. Complications such as reversible bilateral deafness due to spotted fever rickettsioses are infrequently reported in the literature.

**Case Presentation:**

We present a young pregnant female who developed bilateral sensorineural deafness on the fifth day of an acute febrile illness with a maculopapular rash. *Rickettsia conorii* IgG (>1/450) became highly positive, and she received oral azithromycin for ten days with complete resolution of fever and rash. The sensorineural deafness slowly improved over several months.

**Conclusion:**

Sensorineural deafness is a rare complication of spotted fever group rickettsioses. Since usage of doxycycline is controversial in pregnancy, azithromycin gave a favourable recovery of fever. Sensorineural deafness took several months to resolve after completion of antibiotics.

## 1. Introduction

Spotted fevers (SFs) are a group of zoonosis caused by *Rickettsia* spp. They are tick-borne rickettsioses where man becomes an accidental host in the life cycle of rickettsial organism following a tick bite [1–5]. In the broad genus of *Rickettsia*, spotted fever group rickettsiae (SFGR) includes about 15 different species [4].

Except for Rocky Mountain spotted fever (RMSF) the most severe form of SF, neurological manifestations are uncommon in SFGR unless severely complicated with multiorgan involvement [6, 7]. Sensorineural deafness is a rare manifestation where the eighth cranial nerve gets involved in complicated SF [6, 7]. Kularatne et al. in one of the studies carried out in Sri Lanka describing neurological complications in SFGR reported a single patient with sensorineural deafness [6].

SFGR occurring during pregnancy are rarely reported [8, 9]. Since there are no consensus regarding the diagnosis and management of such rickettsiosis during pregnancy [8–10], reporting such successfully treated cases is critical for future decision making.

Spotted fever group rickettsioses are a group of reemerging diseases in Sri Lanka [6, 11–14]. According to the epidemiological data, the reported number of clinically diagnosed rickettsial illnesses is rising [11–13]. Rickettsial illnesses are the cause in a significant proportion of acute febrile illnesses and pyrexia of uncertain origin in the country [11–13]. As a result of high cost, confirmatory tests are not widely available. In Sri Lanka, *R. conorii* antigen is used to detect SFG antibodies using the indirect immunofluorescence antibody (IFA) test. The exact pathogen causing SFGR in Sri Lanka is not properly identified [6]. Therefore, considering the wide cross-reactivity among the pathogens in this group of illnesses [3, 5, 6, 15], we use the broad term “spotted fever group rickettsioses” indicating seropositive rickettsial illness with typical features of SFGR.

We report a pregnant female who was presented with fever and bilateral sensorineural deafness due to infection by SFGR most probably *R. conorii*, which resolved after several months after being treated with a short course of an oral antibiotic.

## 2. Case Presentation

**Figure 1:**
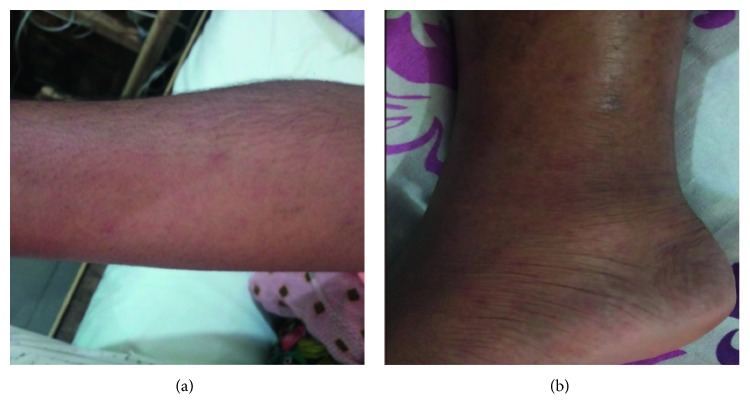
Generalized erythematous maculopapular rash at the (a) upper limb and (b) lower limb.

An otherwise healthy 33-year-old woman in her eighteenth week of pregnancy presented with a five-day history of high spiking fever, generalized body aches with myalgia, and frontal headache. She had noticed a generalized maculopapular rash on the third day of the illness. On the fifth day, she noticed that her hearing got progressively impaired, worst towards the end of the day. She had no seizures, alteration of consciousness, or behavioral changes. On admission to us, she was febrile and had a widespread erythematous maculopapular rash involving the palms and soles but sparing the face (Figure 1). She had no eschar. There were no signs of meningeal irritation. Except for the involvement of the eighth cranial nerve, there was no other cranial nerve involvement. Other focal signs were absent, and the optic fundus was normal. She had no lymphadenopathy or hepatosplenomegaly. She could not recall any history of tick bite. Her immunization was up-to-date, and she was immunized against measles, rubella, and chicken pox in the past.

**Figure 2:**
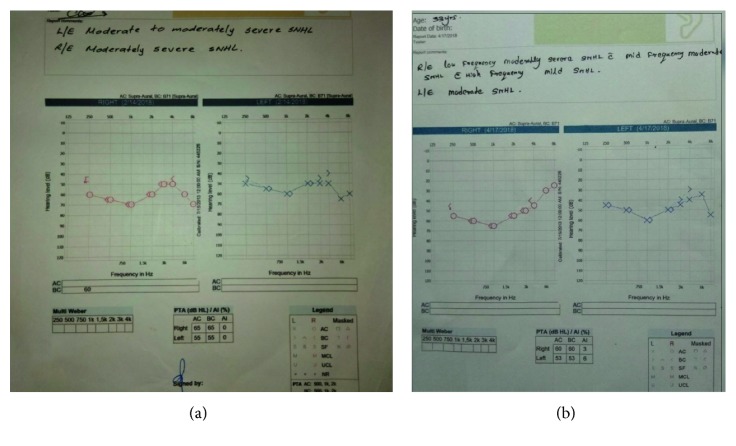
Pure tone audiometry showing bilateral moderately severe sensoneural deafness at presentation (a) and some improvement after one month (b).

Her total white cell count was 3490/*μ*L with 80% neutrophils and 15% lymphocytes. She had a mild thrombocytopenia of 128,000/*μ*L. Her inflammatory markers were elevated including CRP of 120 U/L and ESR of 85 in the 1st hour. Except for a mild transaminitis (AST 58 U/L and ALT 60 U/L), rest of the liver functions were normal. Cerebrospinal fluid (CSF) analysis performed on the 6th day of the illness was normal. The audiometry studies confirmed bilateral sensorineural deafness (Figure 2(a)). Neuroimaging was not performed since the patient was reluctant to undergo computerized tomography or magnetic resonance imaging due to the pregnancy. The virology screens (HIV, HSV, CMV, mumps, measles, and rubella) and serology for syphilis were negative. The Weil–Felix test was positive with a high reactivity of OX19 and OX2 antigens. She received oral azithromycin for a total of 10 days with a presumed diagnosis of rickettsial spotted fever. With this treatment, her fever settled, and her general condition dramatically improved with the maculopapular rash gradually disappearing. The inflammatory markers came down with a CRP of <5 U/L and ESR of 35 mm in the 1st hour at the end of ten days of treatment. Hearing impairment persisted and showed mild gradual improvement after one month (Figure 2(b)). Her diagnosis of spotted fever was serologically confirmed with very high titers of *Rickettsia conorii* IgG (>1/450) after two weeks of the illness. After about five to six months, hearing was restored back to her normal, and by this time, she had an uncomplicated delivery. The baby did not have any physical abnormalities.

## 3. Discussion

Hearing impairment in rickettsiosis can be unilateral or bilateral, and it might resolve within days, months, or sometimes years [7]. When scrub typhus is concerned, nearly one-third of patients are susceptible to develop transient deafness which is considered as a clinical feature, favoring the diagnosis [7, 16, 17]. Since deafness associated with SF is considered rare, this case brings out useful insight into the disease.

SFGR are usually clinically diagnosed owing to their characteristic features of fever, headache, myalgia, and skin involvement [1, 2, 4, 5, 15] which were the prominent features of our patient. Although the clinical presentations of illness due to different organisms of the same species overlap, still clinical signs and symptoms (e.g., rash and other cutaneous findings), epidemiology, laboratory findings, and case fatality rates differ by pathogen, therefore favoring the diagnosis of one disease over the other. Rocky Mountain spotted fever (RMSF) is the commonest spotted fever which is the most virulent form associated with a higher case mortality rate. This disease is endemic in the northern, southern, and Central America and characterized by a purpuric and vasculitic rash [2–4, 10]. Mediterranean spotted fever (MSF) is a more benign form of SFGR and is endemic to Southern Europe, North Africa, and Central Asia [2, 3, 5, 7]. This disease is characterized by a maculopapular rash. SFGR caused by *R. africae* is another commonly discussed illness which comes under SFGR, though it is frequently spotless and is confined to Southern Africa [2, 5]. Considering the clinical features including the benign nature of the illness, except for the 8th cranial nerve involvement, *R. conorii* which causes MSF, a spotted fever group rickettsiosis commonly reported in South Asia [2–5, 15], is the most likely pathogen involved here. This is supported by the strong reactivity of *R. conorii* specific antibodies in our patient's serum. Our patient did not have an eschar or a history of tick bite. According to the literature, an eschar is present only in about 50–75% of cases of rickettsioses [4]. Among different groups of rickettsioses, the incidence of eschar differs. In a study carried out in Sri Lanka, only 1–4% of the SFG-seropositive patients had an eschar while 55–67% of the STG seropositives were detected to have an eschar [11]. This is attributed by the fact that transmission of these diseases is by immature larvae and nymphs, which is likely to go unnoticed [4].

Laboratory diagnosis of SF is not as simple as other bacterial illnesses. Since rickettsiae are a group of fastidious organisms, isolation of these species needs special cell culture techniques which are not widely available [3, 4, 10, 14, 15]. Detection of antibodies against rickettsial antigens using IFA is the currently recommended confirmatory test [3, 4, 10, 14]. The diagnosis is usually based on a single high titer of IgM with a fourfold or greater increase in the IgG titer in samples collected at appropriately timed intervals [10]. A single IgG titer which is above the set cut off for a particular geographical area is also recommended to diagnose acute rickettsioses in the literature [5, 6, 10, 14]. Centers for disease control (CDC) and prevention in one of its Morbidity and Mortality Weekly Report (MMWR) in May 2016 suggest a single IgG titer ≥1/65 in the presence of a compatible clinical illness as a supportive tool to diagnose acute rickettsioses [10] although a fourfold rise is the gold standard. In a clinical study conducted in Sri Lanka, Premaratna et al. recommended a single IgG titer ≥1/256 to be diagnostic for acute SFG infections provided that the sample was obtained after 7 days of clinical illness [14], and this recommendation was applicable to our patient. In many previous studies carried out in Sri Lanka, an IgG titer of ≥1/256 was used as a case-defining tool [6, 11–13]. Therefore, with very high titers of >1/450, we considered this as an acute illness with the compatible clinical and epidemiological background. The positive Weil–Felix test which is historically considered as reliable in the diagnosis of rickettsiosis though it lacks sensitivity and specificity [1–3, 15] provided us the initial guidance for further investigations. In the Weil–Felix reaction, *Proteus vulgaris* OX2 antigen reacts strongly with sera from persons infected with SFG rickettsiae and OX19 antigen reacts with antibodies against both the SFG rickettsiae as well as TG rickettsiae. This is in contrast to the reaction in scrub typhus, where agglutination occurs with *Proteus mirabilis* OXK antigen [2, 3, 15, 18]. This evidence was further reinforced by our patient's clinical picture which was more suggestive of SFG rather than STG rickettsioses which was one of our other differential diagnoses. Apart from the deafness, the patient was apparently well. And the skin involvement occurred fairly early in the illness with remarkable involvement in limbs including the palms and soles and was one of the predominant clinical features in the absence of other organ involvement. Scrub typhus is supposed to cause a more prominent inflammatory response, therefore is more likely to present a more severe clinical picture [15]. Maculopapular rash that occurs in scrub typhus is more prominent in the trunk and usually occurs towards the latter part of the illness, and frequently goes unnoticed [2]. According to the local data, the absence of an eschar was also in favor of SFG than STG rickettsiosis [11, 12, 15]. Considering the above facts, we first got done *R. conorii* antibodies which were highly positive. Although cross-reactivity is frequently reported among organisms in the same group of SFG and to some extent with the typhus group, such cross-reactivity is not discussed with regard to STGR [18, 19]. This may be due to the fact that *Orientia tsutsugamushi* the organism responsible for scrub typhus bears several differences to the other species in the same genus including the genetic and antigenic composition [3, 18]. Therefore, we opted not to perform STG antibodies considering the additional cost, likelihood of being less informative in a background where the clinical and laboratory data were strongly suggestive of SFGR and also the unavailability of the test within the country during that period. The local epidemiological data also favor SFG over STG rickettsioses. An islandwide hospital-based study carried out by Liyanapathirana and Thevanesam gives the most important epidemiological data related to serologically confirmed rickettsioses in Sri Lanka. According to the above study, Hambantota District, Southern Province in Sri Lanka, from where our patient was presented, is among the districts where spotted fever group rickettsioses were commonly reported [11]. Thrombocytopenia and slight elevations in hepatic transaminases [1, 3, 5, 10, 15] were also nonspecific, but supportive abnormal lab tests in rickettsioses which our patient had.

In suspected cases of rickettsiosis including SF, empirical treatment is recommended even before laboratory confirmation. The treatment of choice is doxycycline, 100–200 mg/day, given for 1–7 days [1, 8, 10, 15] depending on the severity of the disease. There are many concerns about the use of tetracyclines during pregnancy. Staining of primary teeth and musculoskeletal abnormalities are the potential complications that the fetuses could develop. Acute fatty liver of pregnancy is the concern about the mother [8–10]. Since these adverse effects were associated with older tetracyclines, whether they are applicable to newer derivatives, such as doxycycline, is debatable. An expert review on the above issue concluded that doxycycline when used in therapeutic doses is unlikely to cause substantial teratogenicity [10]. But the above conclusion was solely made upon observations since there are no controlled studies carried out to assess whether doxycycline is safe enough to be used during pregnancy. Therefore, the currently available data are insufficient to conclude that no risk exists. Maternal hepatic toxicity caused by doxycycline is not within the published data [10]. Concerning all these facts, treatment of SF during pregnancy becomes an individually tailored decision. Recent literature recommends macrolides, namely, clarithromycin, josamycin, and azithromycin for pregnant females as an alternative treatment to doxycycline [8–10]. When there are complications with other organ involvement, a slightly prolonged course of antibiotics is recommended [10]. Taking all these facts into account, we treated our patient with azithromycin for ten days with which she recovered.

## 4. Conclusion

Sensorineural deafness is a rare complication of SF which is reported in a limited number of case reports. Although doxycycline is the recommended treatment in SF rickettsioses, in pregnancy, azithromycin may be preferred, and it led to clinical improvement though sensorineural deafness took several months to recover completely.

## Abbreviations

SF: Spotted fever
SFG: Spotted fever group
SFGR: Spotted fever group rickettsioses
RMSF: Rocky Mountain spotted fever
IFA: Indirect immunofluorescence antibody
AST: Aspartate aminotransferase
ALT: Alanine transaminase
CRP: C-reactive protein
CSF: Cerebrospinal fluid
HIV: Human immunodeficiency virus
HSV: Herpes simplex virus
CMV: Cytomegaly virus
MSF: Mediterranean spotted fever
IgG: Immunoglobulin G
TG: Typhus group
STG: Scrub typhus group.
